# A Review of Sustainability Enhancements in the Beef Value Chain: State-of-the-Art and Recommendations for Future Improvements

**DOI:** 10.3390/ani7030026

**Published:** 2017-03-22

**Authors:** Danielle Maia de Souza, Ruaraidh Petre, Fawn Jackson, Monica Hadarits, Sarah Pogue, Cameron N. Carlyle, Edward Bork, Tim McAllister

**Affiliations:** 1 Department of Agricultural, Food & Nutritional Science, University of Alberta, Edmonton, AB T6G 2P5, Canada; cameron.carlyle@ualberta.ca (C.N.C.); ebork@ualberta.ca (E.B.); 2 Agriculture and Agri-Food Canada, Lethbridge Research Centre, Lethbridge, AB T1J 4B1, Canada; sarah.pogue@uleth.ca; 3 Global Roundtable for Sustainable Beef, Dorpsstraat 45, Geesteren 7678 AV, Overijssel, The Netherlands; ruaraidh@petre.nl; 4 Canadian Roundtable for Sustainable Beef, 180-6315 8th Street NE, Calgary, AB T2E 7H7, Canada; jacksonf@cattle.ca (F.J.); hadaritsm@cattle.ca (M.H.); 5 Department of Geography, University of Lethbridge, Lethbridge, AB T1K 3M4, Canada

**Keywords:** sustainable beef, beef industry, sustainability, continuous improvement, life cycle assessment

## Abstract

**Simple Summary:**

To better address consumer concerns, the beef sector is working on strategies to enhance the sustainability of all aspects of the beef supply chain. Among these strategies are (1) the development of science-based frameworks and indicators capable of measuring progress at all stages of beef production; (2) the engagement of different stakeholders along the beef supply chain at regional and global levels; and (3) the improvement of communication among stakeholders and transparency towards consumers. Progress on these three fronts was presented during the 2nd Global Conference on Sustainable Beef, hosted by the Global and Canadian Roundtables for Sustainable Beef. During the event, there was a clear understanding that the beef industry is substantially advancing efforts to continuously improve its sustainability, both at regional and global levels, by developing assessment frameworks and indicators to measure progress. However, it is also clear that the beef sector has a need to more clearly define the concept of beef sustainability, strengthen cooperation and exchange of information among national roundtables for sustainable beef, as well as improve the flow of information along the supply chain. An improved transparency in the beef sector will help consumers make more informed decisions about food products.

**Abstract:**

The beef sector is working towards continually improving its sustainability in order to achieve environmentally, socially and economically desirable outcomes, all of which are of increasing concern to consumers. In this context, the Global Roundtable for Sustainable Beef (GRSB) provides guidance to advance the sustainability of the beef industry, through increased stakeholder engagement and the formation of national roundtables. Recently, the 2nd Global Conference on Sustainable Beef took place in Banff, Alberta, Canada, hosted by the GRSB and the Canadian Roundtable for Sustainable Beef. Conference attendees discussed the various initiatives that are being developed to address aspects of beef sustainability. This paper reviews the main discussions that occurred during this event, along with the key lessons learned, messages, and strategies that were proposed to improve the sustainability of the global beef industry.

## 1. Introduction

The beef sector is expending considerable effort to improve its sustainability, through quantifying specific impacts of beef production practices on various social, economic and environmental attributes. This information is then being used to develop practices and implement strategies that assist the industry in maintaining its social license. Consumers have begun to engage more actively in the topic of agricultural sustainability, questioning the origin of their food and how it is produced, and this has had cascading effects throughout the value chain of individual commodities, including beef. To address consumer concerns and advance sustainability efforts, multiple initiatives engaging different stakeholders along the beef value chain have developed science-based frameworks to advance science and promote improvements at all stages of beef production. For instance, decision-support tools, such as life cycle assessment (LCA), have been applied to quantify potential environmental impacts from beef production [1,2,3].

The implementation of science-based metrics is fundamental to identifying strategies to improve and communicate the performance of beef production systems to different stakeholders [4]. This is especially important given that many beef production systems, particularly cow/calf production, are very heterogeneous, with pronounced differences in management practices (e.g., animal husbandry and grazing systems), breeds, and feed management [5,6].

**Figure 1:**
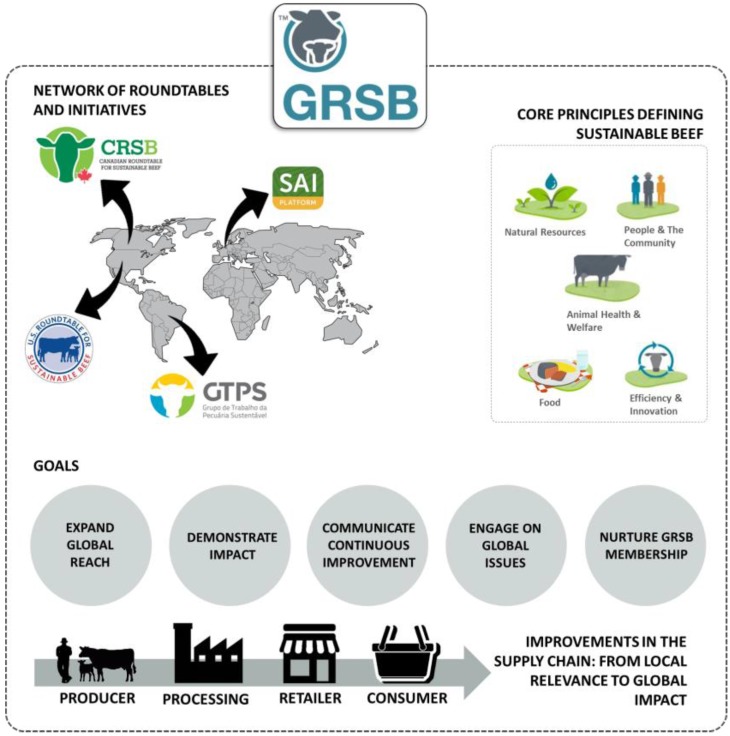
Schematic representation of the multi-stakeholder initiative, Global Roundtable for Sustainable Beef (GRSB), and associated national roundtables and initiative: (1) Canadian Roundtable for Sustainable Beef (CRSB); (2) US Roundtable for Sustainable Beef (USRSB); (3) Brazilian Roundtable on Sustainable Livestock (GTPS); and (4) Sustainable Agriculture Initiative (SAI) Platform. The figure shows GRSB core principles defining sustainable beef, and sustainability goals.

The Global Roundtable for Sustainable Beef (GRSB), a multiple stakeholder initiative formed in 2012 to advance the sustainability of the beef value chain, reached consensus on a global definition of sustainable beef as well as five broad principles within which sustainability is categorized (Figure 1). Sustainable beef is defined as a socially responsible, environmentally sound and economically viable product that prioritizes five principles: (1) natural resources; (2) people and the community; (3) animal health and welfare; (4) food; and (5) efficiency and innovation [7,8].

With the goal of increasing stakeholder engagement and disseminating strategies to continuously improve sustainability of the beef industry around the globe, the GRSB and the Canadian Roundtable for Sustainable Beef (CRSB) hosted the 2nd Global Conference on Sustainable Beef (GCSB), in Banff, Alberta, Canada, on 4–7 October 2016. Nearly 220 attendees from 15 countries, representing beef value chain stakeholders, environmental and social NGOs, governments and academia, discussed the various ways sustainability is being advanced in the beef industry. The event was organized into six breakout sessions, preceded by a keynote speech and a roundtable discussion. The sessions entailed six different topics: (1) sustainability research review; (2) assessing the sustainability of the global beef value chain; (3) business commitments to sustainability; (4) connecting consumers and sustainability; (5) sustainability on the ground from a producer’s perspective; and (6) advancing zero (i.e., gross and net) deforestation within beef production. Here we report on these six breakout sessions and keynote speeches to summarize the current state-of-the-art knowledge in beef production sustainability, and provide recommendations for future directions.

## 2. Keynote Speech and Roundtable Discussion: A Greater Transparency towards Consumers through Measured Progress

The keynote speech drew attention to multiple drivers influencing human demand for high-quality protein, of which beef is a significant source. In recent years, health concerns associated with beef consumption, animal welfare, food safety and environmental protection have led some members of the public to shift their diet towards plant-based protein sources. However, it was noted that some of the information that promoted this transition did not consider a detailed assessment of the beef supply chain. The keynote’s primary message was the need for increased clarity in the information provided to the final consumer so that informed dietary choices can be made. Therefore, market trends and consumer values should be fully considered during the development of a path forward to support social license.

Following the keynote speech, the roundtable discussion addressed the concept of sustainability within the beef industry and the importance of the beef sector in directly conveying a transparent message to the consumer about progress in improving sustainability. Different roundtables and initiatives (e.g., from Canada, Brazil, the United States, and Europe) showcased their work at the national level and their progress towards a more sustainable beef supply chain. Among the discussions, there was a clear focus on the need to measure progress via well-defined sustainability indicators, such as enhanced animal welfare, worker safety and water resources. In Canada, the CRSB’s National Beef Sustainability Assessment on Strategy project is assessing the environmental, social and economic sustainability performance of the Canadian beef industry, from primary beef production all the way through to retail and consumption. The Brazilian Roundtable on Sustainable Livestock (GTPS) is working to build consensus in the entire value chain, based on sound agricultural and livestock management practices, while guaranteeing continuous improvement and compliance with environmental stewardship legislation. The US Roundtable for Sustainable Beef (USRSB) has designed priority indicators, including metrics for water resource use, animal welfare and greenhouse gas emissions, to ensure industry progress towards advancing and communicating continuous improvement in sustainable beef production. The USRSB is also working on the implementation of an assurance framework, which is comprised of educational and training programs for producers and self-assessment through LCA. In Europe, the Sustainable Agriculture Initiative (SAI) Platform has demonstrated the importance of harmonizing practices, reducing complexity and engaging different actors along the supply chain, to improve sustainable agriculture. In particular, a beef working group composed of different stakeholders (i.e., beef processors, retail, food manufacturers and farmer organizations) has been working to improve sustainability of the beef industry and convey a more positive message about the sector’s European activities. The Australian beef industry, although not under the umbrella of the GRSB, is working to identify several priority areas (i.e., welfare, economic, environment, community) and indicators to reflect the current practices of the Australian beef industry.

## 3. Breakout Sessions

### 3.1. Sustainability Research Review

There has been a marked increase in the amount of protein consumed by the world’s human population [9]. Future scenarios point to further increases in the demand for meat by 2050, mainly sustained by augmented supply and demand from developing countries [10]. As a result, there are important questions around how the livestock sector will meet this demand in an efficient and sustainable manner. For example, significant challenges are expected to be associated with maintaining animal health within intensive production systems, and increased environmental pressures are anticipated from greater animal, and resulting manure, densities. Solutions will center on strategies involving innovation along the whole supply chain, including improved engagement with primary producers and socio-economic considerations. As a result, the need to define key performance indicators and associated metrics capable of informing progress, while being output oriented, science-based and transparent, will be critical.

In the context of defining metrics for sustainability, the Food and Agriculture Organization of the United Nations (FAO) Livestock Environmental Assessment and Performance Partnership (LEAP) reported on its key role in fostering progress to improve the sustainability of the entire livestock sector [11]. By recognizing the complexity of different production systems, multiple stakeholders developed guidelines [12], as well as science-based methodologies to assess the impacts of livestock supply chains, such as a harmonized approach to assess the environmental performance of large ruminant supply chains [13]. As an outcome, a series of documents containing recommendations have been produced, and work is ongoing focusing on identifying knowledge gaps and research needs in areas such as water use, biodiversity [14,15], and soil carbon.

### 3.2. Assessing the Sustainability of the Global Beef Value Chain

A primary focus of the presentations in this breakout session was the improvement of the research metrics and indicators that define sustainability within the beef industry. The importance of assessing the contribution of grazing lands and related management practices to the maintenance of biodiversity and the supply of ecosystem services beyond their role as feed for beef (e.g., carbon storage and sequestration, water and soil quality) were key points of discussion.

Results of the CRSB’s National Beef Sustainability Assessment and Strategy project [16] were presented in detail. This assessment strives to identify the impacts and benefits of cattle production in different fields, such as climate change, water, and land use [16]. The study showed that, although regional differences in beef production processes and management practices exist [8], Canada is an efficient beef producer with regard to greenhouse gas emissions and blue water use per kilogram of beef produced. Additionally, it was noted that several major research initiatives are underway in Canada that involve researchers from the University of Alberta, University of Lethbridge, Agriculture and Agri-Food Canada and organizations such as the Alberta Biodiversity Monitoring Institute, in order to assess the relationship between beef production and other ecosystem services. This includes the ‘Biodiversity Assessment of Alberta’s Beef Industry’ project, led by the Alberta Biodiversity Monitoring Institute (ABMI), that is assessing cattle production impacts on biodiversity, by means of regionalized life cycle impact assessment [17]. The aim is to assess differences in land use and grazing management practices, and their subsequent impacts on biodiversity and ecosystem services at the regional level, using indicators and a virtual farm system approach.

### 3.3. Business Commitments to Sustainability

During this breakout session, different companies reported on the work they are undertaking to implement sustainability within their businesses, and each provided insight into how the efforts led by the roundtables are being put into practice within their beef supply chains. Improvements included a reduction in the use of antibiotics shared by the cattle and human health sectors, and the provision of more information to consumers about the manner in which food is produced, including how cattle are handled. Regarding the use of antibiotics, there is the need to balance the use of antibiotics with the increased demand for food, the need to ensure human health and nutrition, and the maintenance of animal and environmental health.

The importance of stronger engagement of different stakeholders in the supply chain was emphasized as a way to ensure the quality and availability of products. Instruments to help measure improvement at the farm level, and to manage data and integrate other systems and tools, have been employed to enable farmers to better understand how advances in technology can help solve sustainability issues.

### 3.4. Connecting Consumers and Sustainability

There remains a clear need for better communication among different players in the beef supply chain, which includes the consumer, to ensure that the most accurate and up-to-date information about the beef industry is provided. In this context, different organizations have been working to build consumer trust by creating a closer dialogue among different players in the supply chain and coordinating strategies to maintain or enhance public trust. For that, an increased understanding of consumers’ expectations is a key step to establishing more effective communication through shared values and shared content through information compiled in a single platform or website.

As an example, some consumers are advocating to raise animal welfare and health standards, even if it results in a higher cost of beef. Industry can increase consumer trust by using clear labels and assurance schemes that are backed by brand values, which may ensure a greater degree of transparency and better communication of its products. The consideration of traceability aspects along the entire beef supply chain, from farm to fork, is essential to frame the debate around beef sustainability.

### 3.5. Sustainaiblity on the Ground: A Producer’s Perspective

A group of producers from different continents shared their experience in aligning their production systems with environmental protection and conservation programs. For instance, practices such as integrated agriculture (i.e., shared areas with livestock, silviculture and crop areas) in Brazil have been shown to improve land quality and promote biodiversity. In Paraguay, a number of strategies have been implemented in the Southern Chaco region of the country. These include improved pasture management, the incorporation of a backgrounding stage, a breeding program for improved feed efficiency, and community involvement, all of which are aimed at increasing the efficiency of beef production and improving economic performance, reducing the environmental footprint, and achieving social inclusiveness. In Europe, the focus on animal welfare, genetic selection and management of herds on the land (e.g., limiting access to waterways) have identified strategies to reduce the impacts of cattle on pasture and riparian areas, thereby reducing erosion and water contamination.

The idea of continuous improvement was recognized as a way to ensure sustainability for future generations (e.g., soil and water quality, animal welfare), an objective that requires relevant metrics to track processes. Overall, it was noteworthy that when establishing conservation programs, there is the need to set specific goals and identify clear natural habitat conservation targets. Although there are opportunities, this task does not go unchallenged, as issues such as the alignment of beef production practices with environmental goals need to be understood, including how these relationships change with adaptive management strategies.

### 3.6. Advancing Zero Deforestation within Beef Production

The concept of zero deforestation encompasses specific commitments to deforestation reduction targets by different governments, institutions, and/or organizations [18]. Representatives from Brazil, Paraguay and Argentina have demonstrated how different management programs and projects, sometimes driven by the need to comply with Federal and State regulations on forest conservation, have reduced gross and net deforestation in natural areas. The Brazilian Roundtable on Sustainable Livestock elaborated on the Novo Campo Program, a voluntary program aimed at improving sustainable livestock practices in the Amazon region. In order to participate in the program, producers need to comply with a set of requirements related to animal welfare, social responsibility, and environmental conservation. For example, producers are required to conserve remaining natural areas and restore degraded ones, and comply with the Brazilian Forest Code. The program carries out an assessment of forest management practices on each farm, and designs farm-specific projects along with resources for capacity building. In Paraguay, the Ministry of Agriculture and Livestock presented the national Breeding Program, which involves the genetic improvement of species and the replacement of native grass species with more productive Pangola grass to improve the efficiency of beef production. The key objective of the program is to increase productivity per unit area in order to decrease the pressure to convert remaining forests and savannah to use for beef production. The Argentinian Ministry of Agroindustry is working to provide guidance on the establishment of forest management practices that are integrated with cattle ranching at the farm level (e.g., regeneration of natural areas, and fire prevention and control).

Some companies are also making efforts to reduce deforestation, acting on responsible sourcing of their products (e.g., beef, coffee, palm oil and fish) and aiming at the use of only fiber-based certified/recycled packaging. The Nature Conservancy, an organization addressing threats to ecological diversity, is supporting the implementation of a model for sustainable livestock production in Brazil, emphasizing the need for producers to comply with environmental regulations, intensify production, track their production and achieve zero deforestation. A pilot project has been implemented in the municipality of São Félix do Xingu, involving different stakeholders including producers, government and industry. The project acts on social (e.g., capacity-building), economic (e.g., a low-carbon agriculture credit program), and environmental aspects, and has led to an 83% reduction in deforestation rates within the area over the last eight years, while promoting the restoration of large tracks of pasture.

## 4. Discussion

After the GCSB concluded, a participant survey was undertaken to assess the main impressions of the attendees at the event. There was general agreement on three main items. First, there was a clear understanding that substantial progress has been made on the continuous improvement and sustainability of the beef industry at global and national levels. For instance, the progress achieved by each of the national roundtables was identified as a noteworthy effort to increase stakeholder engagement and define assessment frameworks through LCA and the establishment of indicators to measure progress. However, some participants emphasized the importance of enhancing the exchange of regional experiences, as well as the significance of producing more concrete results at the local scale through more tangible and demonstrable examples of improvement and measurable impact. Strengthened cooperation among the national roundtables will help achieve this aim by filling existing gaps through shared knowledge, as well as by improving the industry’s ability to more effectively communicate information to consumers. Secondly, participants perceived the need to more clearly define the concept of ‘sustainable beef’ to be used in the work developed across different nations. Although it is recognized that sustainability practices will differ among individual beef production systems, a more solid and clearer definition would help align national strategies and indicators, and thereby replicate successful experiences in different regions of the world. Finally, from the standpoint of the producers, the need to bridge the gap between the information conveyed along the beef value chain, from the primary producer to the final consumer, was seen as a critical component of any sustainability plan. One way to potentially tackle this problem is the development of strategies to more actively engage producers in national efforts and roundtable discussions, as well as in the activities of the GRSB. It is clear that sustainability is an evolving metric and will continually require adjustment as productions systems adapt and the needs and desires of consumers continue to evolve.

## 5. Conclusions

The beef sector has presented advances on strategies, such as assessment frameworks and indicators aimed at improving the sustainability of its supply chain. National roundtables have strongly contributed to this progress. However, some challenges remain and there is still work to be done for the beef sector to advance its sustainability. The fast-paced escalating environmental concerns, aligned with social and economic pressures, will continue to motivate the beef industry to re-define various aspects of its supply chain. First, better management of genetic resources (i.e., beef and feed crops), at the farm, regional and national levels, integrated with science-based approaches and research will be needed to increase nutrient use efficiency, and improve the environmental performance of beef production systems. Therefore, an enhanced integration of knowledge along the supply chain is needed. Second, decisions at the political level are also foreseen such as recent changes in legal use of shared-class antibiotics for enhancing growth and feed efficiency in the United States. The beef industry will need to develop strategies to overcome related difficulties and provide more rapid responses to antimicrobial concerns. Finally, the business commitment to sustainability at the primary production level should be enhanced, by providing producers with appropriate frameworks and strategies to respond to aspects such as economic risks that may be linked to factors such as climate change or shifts in consumer demand. In addition, improved platforms for data sharing should allow a two-way communication between national roundtables and farmers. On one hand, these platforms would allow for more effective identification of challenges and opportunities encountered at the farm level while providing a better understanding of knowledge generated at regional and national scales.

One area that will become increasingly important is transparency and information sharing throughout the beef supply chain. Integration of effective traceability systems into supply chain management, as well as the use of technological innovations such as remote sensing and computerized information systems, will enable more rapid responses to consumer inquiries and will aide in ensuring the integrity of the beef supply chain [19].

## References

[1] Beauchemin K.A., Henry Janzen H., Little S.M., McAllister T.A., McGinn S.M. (2010). Life cycle assessment of greenhouse gas emissions from beef production in western Canada: A case study. Agric. Syst..

[2] De Vries M., van Middelaar C.E., de Boer I.J.M. (2015). Comparing environmental impacts of beef production systems: A review of life cycle assessments. Livest. Sci..

[3] Legesse G., Beauchemin K.A., Ominski K.H., McGeough E.J., Kroebel R., MacDonald D., Little S.M., McAllister T.A. (2016). Greenhouse gas emissions of Canadian beef production in 1981 as compared with 2011. Anim. Prod. Sci..

[4] Pelletier N. (2015). Life Cycle Thinking, Measurement and Management for Food System Sustainability. Environ. Sci. Technol..

[5] Herrero M., Havlík P., Valin H., Notenbaert A., Rufino M.C., Thornton P.K., Blümmel M., Weiss F., Grace D., Obersteiner M. (2013). Biomass use, production, feed efficiencies, and greenhouse gas emissions from global livestock systems. Proc. Natl. Acad. Sci. USA.

[6] Sheppard S.C., Bittman S., Donohoe G., Flaten D., Wittenberg K.M., Small J.A., Berthiaume R., McAllister T.A., Beauchemin K.A., McKinnon J. (2105). Beef cattle husbandry practices across Ecoregions of Canada in 2011. Can. J. Anim. Sci..

[7] Global Roundtable for Sustainable Beef (GRSB) (2015). Principles and Criteria for Defining Global Sustainable Beef.

[8] Global Roundtable for Sustainable Beef (GRSB) (2016). Strategic Plan 2016–2021.

[9] Tilman D., Balzer C., Hill J., Befort B.L. (2011). Global food demand and the sustainable intensification of agriculture. Proc. Natl. Acad. Sci. USA.

[10] Rosegrant M.W., Cline S.A. (2003). Global food security: Challenges and policies. Science.

[11] Food and Agriculture Organization-Livestock Environmental Assessment and Performance (FAO-LEAP) (2016). Developing Sound Tools for Transition to Sustainable Food and Agriculture: Methodological Notes.

[12] Food and Agriculture Organization-Livestock Environmental Assessment and Performance (FAO-LEAP) (2016). Environmental Performance of Animal Feed Supply Chains: Guidelines for Assessment.

[13] Food and Agriculture Organization-Livestock Environmental Assessment and Performance (FAO-LEAP) (2016). Environmental Performance of Large Ruminant Supply Chains: Guidelines for Assessment.

[14] Food and Agriculture Organization-Livestock Environmental Assessment and Performance (FAO-LEAP) (2016). Principles for the Assessment of Livestock Impacts on Biodiversity.

[15] Teillard F., Maia de Souza D., Thoma G., Gerber P.J., Finn J.A. (2016). What does life-cycle assessment of agricultural products need for more meaningful inclusion of biodiversity?. J. Appl. Ecol..

[16] Canadian Roundtable for Sustainable Beef (CRSB) (2016). National Beef Sustainability Assessment and Strategy Summary Report.

[17] ABMI Biodiversity Assessment of Alberta’s Beef Industry. http://abmi.ca/home/projects/applied-research-projects/beef-and-biodiversity.html.

[18] Brown S., Zarin D. (2013). What does zero deforestation mean?. Science.

[19] Dabbene F., Gay P., Tortia C. (2014). Traceability issues in food supply chain management: A review. Biosyst. Eng..

